# Factors influencing treatment outcomes in botulinum toxin–naïve patients with cervical dystonia

**DOI:** 10.3389/fneur.2026.1991239

**Published:** 2026-10-30

**Authors:** András Salamon, Beatrix Kádár, Gabriella Gárdián, Barnabás Klausz, Tamás Mészáros, Mónika Wieland, Péter Klivényi, Dénes Zádori

**Affiliations:** Department of Neurology, Albert Szent-Györgyi Clinical Centre, Albert Szent-Györgyi Faculty of Medicine, University of Szeged, Szeged, Hungary

**Keywords:** botulinum neurotoxin, cervical dystonia, electromyography, outcome, ultrasound

## Abstract

**Background:**

Intramuscular botulinum neurotoxin (BoNT) is currently considered the gold standard treatment for cervical dystonia (CD). Therefore, the primary goal of the study was to explore the associations between eleven variables and patient-reported efficacy of BoNT treatment.

**Methods:**

We retrospectively analysed data from 48 BoNT-naïve CD patients who received ultrasound-guided BoNT treatment between March 2022 and December 2025.

**Results:**

Significant pairwise associations were found only between the ordinal outcome variable and the average dystonic electromyography (EMG) activity of the injected muscles (*p* = 0.025) and the severity of tremulous/jerky movements (*p* = 0.032). A significant interaction between the average dystonic EMG activity of the injected muscles and the status of tremulous/jerky movements was identified (*p* = 0.002). A strong positive association between the average dystonic EMG activity of the injected muscles and the outcome was present only in patients with tremulous/jerky movements (*p* < 0.001). Patients with tremulous/jerky CD were characterized by a longer disease duration (60 vs. 15 months) and less pronounced average abnormal posturing (18.7° vs. 26.8°) compared to those without tremulous/jerky movements.

**Conclusion:**

We found a significant interaction between average dystonic EMG activity of the injected muscles and the presence of tremulous/jerky movements, highlighting the potential clinical relevance of electrophysiological muscle activity in this clinical phenotype. However, our observations should be interpreted cautiously because of the retrospective design and limited sample size.

## 1 Introduction

Dystonia is characterized by sustained or intermittent abnormal movements and/or postures that are typically patterned, repetitive, and may be tremulous or jerky. Symptoms are often triggered or worsened by voluntary movement and may be accompanied by overflow muscle activation (1). In routine adult movement disorder practice, focal dystonia forms, particularly cervical dystonia (CD), account for the greatest clinical burden. Intramuscular botulinum neurotoxin (BoNT) is currently considered the gold standard treatment for CD (2). However, clinical experience indicates that response to BoNT varies substantially among patients, making the identification of factors associated with favourable and unfavourable treatment outcomes an important clinical goal (3).

Previous studies have shown that the therapeutic response to BoNT depends on several factors, among which accurate identification and selection of the dystonic muscles is considered one of the most important (3). Various, often combined approaches, including clinical examination (e.g., palpation and the use of anatomical landmarks), electromyography (EMG), ultrasound (US), and the COL-CAP classification, have been introduced to improve muscle selection and accurate injection targeting (3, 4). However, once the appropriate muscle(s) have been identified, optimal estimation and distribution of the BoNT dose is equally important to maximize clinical benefit while minimizing adverse effects (3, 4). Furthermore, widely used clinical rating scales (e.g., Toronto Western Spasmodic Torticollis Rating Scale (TWSTRS) and Tsui scales) do not always correlate with patient-reported outcome measures – especially in the context of non-motor symptoms (e.g., depression, anxiety, inappropriate expectations regarding treatment benefit)—making the interpretation and comparability of treatment efficacy challenging (5). Neutralizing antibodies against BoNT (primary and secondary immunoresistance) do not seem to play an important role in suboptimal clinical responses (6, 7). An incorrect diagnosis, highly complex dystonic patterns, and secondary structural muscle changes (e.g., fibrosis, contractions) resulting from surgery or repeated BoNT treatments more likely reduce the probability of achieving an optimal clinical outcome (3). The associations of tremulous/jerky movements and the time elapsed between disease onset and the first BoNT injection remains uncertain, with conflicting findings reported across studies (8, 9).

Therefore, the primary goal of this study was to explore the associations between eleven variables and patient-reported efficacy of BoNT treatment. As a secondary goal, the relationship between variables was also analysed.

## 2 Patients and methods

We retrospectively analysed data from 48 BoNT-naïve CD patients who received US-guided BoNT treatment between March 2022 and December 2025. The treatment outcome of their first injection cycle was rated by themselves and reported on the date of the subsequent injection using the following 7-point Likert scale: major benefit (+3), moderate benefit (+2), minor benefit (+1), no effect (0), minor deterioration (−1), moderate deterioration (−2), major deterioration (−3). The patient-reported rating specifically referred to the period during which the patient perceived the effect of the injection. A standardized pre-treatment clinical assessment was performed using the modified Tsui scale. The interval between the first and subsequent injection was almost exclusively 13 weeks (median: 13, minimum: 12, maximum: 14, interquartile range: 0). The effect of the following variables was assessed on outcome: age, sex, disease duration, presence of tremulous/jerky movements, severity of tremulous/jerky movements, mean angular deviation of abnormal posture (calculated as the average of angular deviations measured for all identified COL-CAP forms), total BoNT dose, use of EMG (Dantec Clavis handheld device), average number of injected muscles, average dystonic EMG activity of injected muscles using a semiquantitative grading of 0 (no audible activity), 1 (scarce/occasional audible activity), 2 (frequent but intermittent audible activity), and 3 (almost continuous audible activity), and average thickness indicator of the injected muscles. EMG assessment was available in 42 of the 48 patients. These 42 patients underwent combined US and EMG-guided assessment, while the remaining 6 patients received US-guided injections without EMG assessment. The six patients were not excluded from the overall cohort and were included in analyses that did not involve EMG-derived variables. They were not considered as a separate control group due to low case number, and as the study was not designed to compare EMG-guided with US-only treatment. The grading of EMG activity was based on the consensus of two injectors with considerable expertise in the evaluation and management of patients with CD. The assessors were not blinded to the clinical phenotype. No formal intra-rater or inter-rater reliability assessment was performed in this retrospective study. The average thickness indicator of the injected muscles was calculated as a ratio between the thickness of the injected muscle at the site of injection and that of the contralateral non-injected muscle at the same level. For muscles treated bilaterally, the thickness of the injected muscle was normalized to the mean thickness of the corresponding non-injected muscle measured across the study population. This approach was applied as a pragmatic reference measure in the absence of a contralateral non-injected muscle, recognizing that it does not account for interindividual differences in muscle size. Muscle selection for injection was based primarily on the overall clinical phenotype and examination, including abnormal posture and movements, muscle hypertrophy, and pain. EMG findings, when available, were considered as an additional source of information rather than an independent criterion determining treatment. Accordingly, EMG findings only partially contributed to muscle selection and dose allocation. Furthermore, the potential influence of primary complaints and treatment goals—available in 45 out of 48 patients – on outcome was also analysed. In 16 patients (35.6%), the primary treatment goal was improvement of tremulous/jerky movements. All statistical calculations were performed with the freely available R Project for Statistical Computing (RRID: SCR_001905; R Development Core Team, https://www.r-project.org/). The distribution of continuous variables was checked with QQ plots and the Shapiro–Wilk test where normality of data populations was an underlying assumption. Then the homogeneity of variances was assessed with Levene’s (non-Gaussian distribution) or Bartlett’s (Gaussian distribution) tests in all the cases where this assumption should be met. Pairwise associations between the ordinal outcome variable and continuous or ordinal variables were first explored using Spearman’s rank correlation analyses. The effects of binary variables were explored using the Mann–Whitney-Wilcoxon test. Variables showing at least a trend (*p* < 0.1) were considered for inclusion in a multivariable ordinal logistic regression model as a part of screening approach. As no significant variables were identified in the multivariable ordinal model and model instability was observed when testing interaction terms (likely due to sparse data and quasi-separation), an exploratory linear regression approach was applied to further investigate potential interaction effects. In this model, the ordinal outcome was treated as an approximately continuous variable, and the severity of tremulous/jerky movements was dichotomized (absence vs. presence) to reduce model complexity and improve stability, and an interaction term between the average dystonic EMG activity of injected muscles and the status of tremulous/jerky movements was introduced. The observations were assumed to be independent, as each patient contributed a single measurement. Multicollinearity was assessed using variance inflation factors and was not found to be significant. Other model assumptions were evaluated using graphical diagnostics, including assessment of linearity, homoscedasticity, and the distribution of residuals. These did not indicate major violations, although a slight increase in residual variance at higher fitted values was observed, which was not considered to materially affect the results. Influential observations were identified using influence measures, including Cook’s distance and leverage. Sensitivity analyses were performed by excluding influential observations to assess the robustness of findings. Associations between ordinal or continuous variables were assessed using Spearman’s rank correlation analyses. Group comparisons were performed using the Mann–Whitney-Wilcoxon test (*k* = 2) or Kruskal-Wallis test (*k* > 2) for non-Gaussian or ordinal data, and independent *t*-tests (*k* = 2) for normally distributed continuous variables. Associations between nominal variables were assessed using Fisher’s exact test. Data are presented as median and interquartile range with minimum and maximum values (plus outliers if present). The null hypothesis was rejected when *p*-values were < 0.05. Effect sizes (r for Mann–Whitney-Wilcoxon tests) were also calculated with 95% confidence intervals in case of significant differences to demonstrate the magnitude of differences found.

## 3 Results

The demographics and characteristics of other variables are presented in Table 1. Significant pairwise associations were found only between the ordinal outcome variable and the average dystonic EMG activity of the injected muscles and the severity of tremulous/jerky movements (Spearman’s 𝜌 = 0.35, *p* = 0.025, and Spearman’s 𝜌 = 0.30, *p* = 0.032, respectively). Due to model instability in the ordinal logistic regression analysis, an exploratory linear regression model was applied, explaining a moderate proportion of variance in the outcome (*R*^2^ = 0.34, adjusted *R*^2^ = 0.27, *p* = 0.004). A significant interaction between the average dystonic EMG activity of the injected muscles and the status of tremulous/jerky movements was identified (*β* = 1.95, *p* = 0.002), indicating that the effect of average dystonic EMG activity of the injected muscles differed depending on the presence of tremulous/jerky movements. As shown in Figure 1, a strong positive association between the average dystonic EMG activity of the injected muscles and the outcome was present only in patients with tremulous/jerky movements (Spearman’s 𝜌 = 0.60, *p* < 0.001), whereas no such relationship was observed in those without tremulous/jerky movements (Spearman’s 𝜌 = −0.44, *p* = 0.13). The total BoNT dose, the average number of injected muscles, and the pre-treatment modified Tsui score did not correlate with the outcome (Spearman’s 𝜌 = 0.16, *p* = 0.27, Spearman’s 𝜌 = 0.24, *p* = 0.1, and Spearman’s 𝜌 = 0.063, *p* = 0.67, respectively). There was no significant difference in the patient-reported outcomes between patients whose primary treatment goal was the improvement of tremulous/jerky movements and those with other treatment goals (Mann–Whitney-Wilcoxon test, *p* = 0.182). Sensitivity analyses excluding influential observations yielded consistent results, with the interaction between average dystonic EMG activity of injected muscles and the status of tremulous/jerky movements remaining statistically significant across models. In contrast, the main effect of average dystonic EMG activity of injected muscles was not consistent and lost significance in some models, suggesting that its association with the outcome is conditional on the status of tremulous/jerky movements. Further analyses of associations between variables, including stratification by the status of tremulous/jerky movements (Table 1), demonstrated that disease duration was significantly shorter in patients without tremulous/jerky movements (Mann–Whitney-Wilcoxon test, *p* < 0.001, *r* = 0.552), and the mean angular deviation of abnormal posture was significantly larger in this group (Mann–Whitney-Wilcoxon test, *p* = 0.0012, *r* = 0.469). These findings remained robust in sensitivity analyses.

**Table 1:** The demographics and characteristics of variables grouped by the presence or absence of tremulous/jerky movements.

Variable	Total (*n* = 48)	Tremulous/jerky (*n* = 32)	Non-Tremulous/jerky (*n* = 16)	*p* value
Age at injection (years)	53.6 (29–78, ±10.9)	55.1 (29–78, ±11.8)	50.6 (38–66, ±8.3)	0.18
Sex (M/F)	9/39	4/28	5/11	0.14
Duration (months)	48 (2–672, 97.5)	60 (2–672, 135)	15 (2.5–168, 15)	< 0.001
Severity of tremulous/jerky movements (on a 0–3 Likert scale)	1 (0–3, 1)	1 (1–3, 1)	0 (0–0, 0)	n.a.
Mean angular deviation of abnormal posture (°)	21.4 (6.7–50, ±10.9)	18.7 (6.7–50, ±8.7)	26.8 (15–42.5, ±7.4)	0.0012
BoNT dose (abobotulinum toxin unit)	368.6 (75–650, ±117.3)	349.6 (75–650, ±129.6)	406.5 (200–525, ±78.0)	0.11
Use of electromyography	42	29	13	0.39
Average number of injected muscles	4 (1–8, 2)	4 (1–8, 2)	4 (2–5, 0.25)	0.359
Average dystonic electrophysiological activity of injected muscles (on a 0–3 Likert scale)	1.2 (0–2.5, ±0.6)	1.35 (0–2.5, ±0.64)	0.98 (0–1.6, ±0.55)	0.084
Average thickness indicator of the injected muscles	1.27 (0.45–2, ±0.3)	1.27 (0.66–1.79, ±0.24)	1.27 (0.45–2, ±0.34)	0.93
Treatment outcome (on a − 3-3 Likert scale)	2 (−2–3, 2)	2 (0–3, 2)	1 (−2–3, 1.25)	0.17

Data are presented as mean (minimum-maximum, standard deviation) or median (minimum-maximum, interquartile range). To reveal the differences in data count, Fisher’s exact test was applied, otherwise the independent *t* test was used for group comparisons, except disease duration, mean angular deviation of abnormal posture, average number of injected muscles, and treatment outcome, where the Mann–Whitney-Wilcoxon test was utilized. BoNT, botulinum neurotoxin; F, female; M, male; n.a., not applicable.

**Figure 1:**
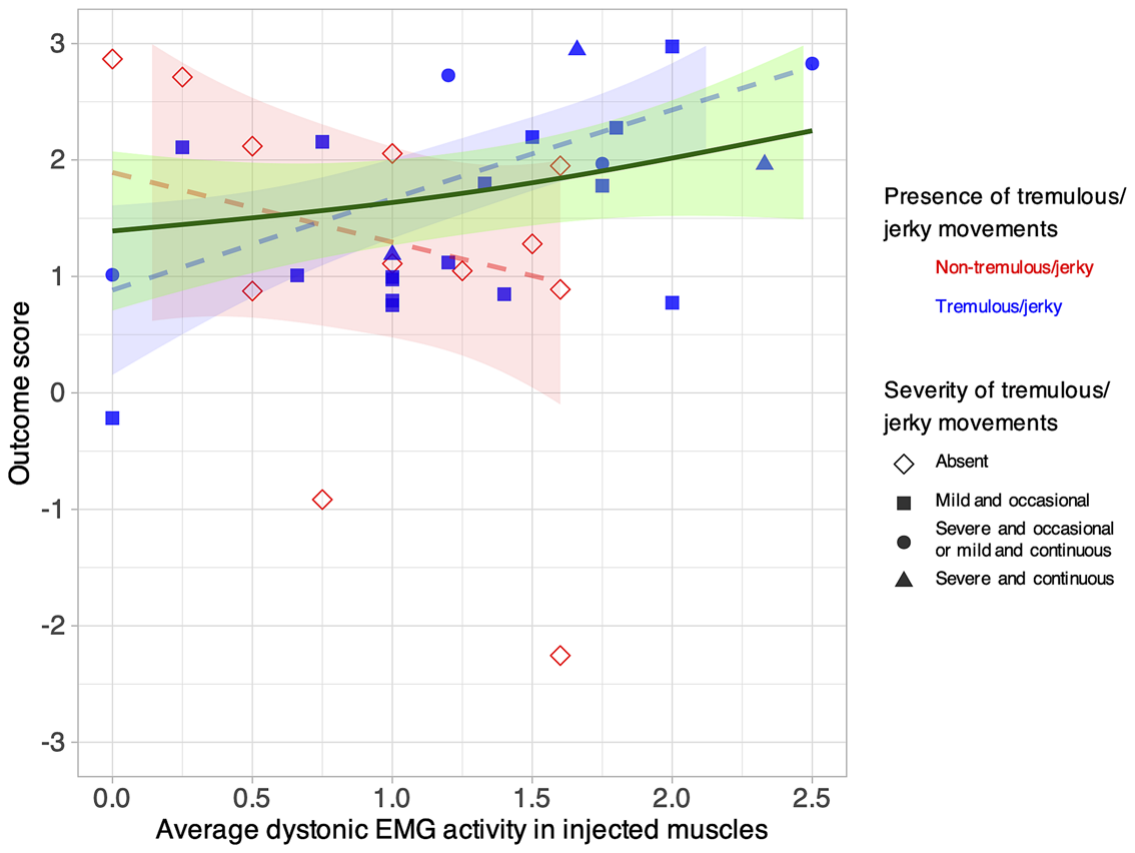
Effect of average dystonic EMG activity of injected muscles on treatment outcome. A significant positive correlation was present (solid green general additive model-based regression curve with 95% confidence interval; Spearman’s *𝜌* = 0.35, *p* = 0.025), which was more prominent in the presence of tremulous/jerky movements (blue dashed regression curve with 95% confidence interval; Spearman’s *𝜌* = 0.60, *p* < 0.001) and was absent in CD patients without tremulous/jerky movements (red dashed regression curve with 95% confidence interval; Spearman’s *𝜌* = −0.44, *p* = 0.13). CD, cervical dystonia; EMG, electromyography.

## 4 Discussion

Identification of factors influencing treatment response to BoNT in patients with CD is challenging. Therefore, to avoid the confounding effects of previous BoNT treatment and the limitations of conventional clinical rating scales, we retrospectively assessed treatment response using a patient-reported outcome measure in BoNT-naïve patients.

Based on our findings, patients with CD may be stratified into two clinically distinct groups according to the presence or absence of tremulous/jerky movements. Patients with tremulous/jerky movements demonstrated a strong positive association between average dystonic EMG activity and patient-reported treatment response, whereas no such relationship was observed in patients without tremulous/jerky movements.

Nijmeijer et al. (10) investigated intermuscular EMG coherence and EMG autospectral analysis as objective neurophysiological tools for identifying dystonic muscles. They found that patients with tremulous or more rhythmic/phasic dystonic activity exhibited prominent low-frequency (4–7 Hz) intermuscular coherence, with synchronized bursting activity and clinically manifest dystonic tremor potentially representing a continuum. In contrast, in patients without pronounced tremor, the most discriminative electrophysiological abnormality was a reduction or absence of the 8–14 Hz auto spectral peak in dystonic splenius muscles, an effect that remained evident after exclusion of the two patients with pronounced tremor. This distinction between tremulous/jerky and posture-predominant phenotypes may be relevant to our findings, as the significant interaction between average dystonic EMG activity and the presence of tremulous/jerky movements may indicate that the electrophysiological characteristics of dystonic muscles differ according to the predominant clinical phenotype.

The total BoNT dose and the average number of injected muscles did not correlate with the patient-reported outcome, suggesting that these treatment-related factors were unlikely to have substantially influenced the observed associations. Furthermore, the modified Tsui score also did not correlate with the outcome, suggesting that baseline objective disease severity was not a major determinant of the patient-reported therapeutic outcome. Individual treatment goals may influence patients’ perceptions of treatment efficacy and satisfaction. In our cohort, however, we found no significant difference in patient-reported outcomes between patients whose primary treatment goal was to improve tremulous/jerky movements and those with other treatment goals.

As secondary findings, in agreement with Merola et al. (8) and Pandey et al. (9), patients with tremulous/jerky CD in our cohort had a significantly longer disease duration and less pronounced abnormal posturing, as reflected by a smaller mean angular deviation of the abnormal head posture. Similarly, Merola et al. reported a longer disease duration (18.9 vs. 13.9 years) and lower TWSTRS severity scores (16.4 vs. 20.3) in patients with tremor-dominant CD, while Pandey et al. also observed a significantly longer disease duration (11.8 ± 8.7 vs. 9.5 ± 8.9 years) and a predominance of simpler CD phenotypes in patients with dystonic head tremor.

Based on the results detailed above, it seems that, in the tremulous/jerky phenotype, which represents a more dynamic manifestation of dystonia, rhythmic or intermittent dystonic muscle activation could be more readily detected and quantified using a small, handheld EMG device, offering a practical and readily accessible approach in routine clinical practice without the need for a more complex EMG setup. This may facilitate the targeting and selection of dystonic muscles and potentially contribute to an improved patient-reported treatment response. In contrast, in patients without prominent dynamic muscle activity, who exhibited greater postural angular deviation, handheld EMG device may provide less additional information for treatment planning, as the predominantly tonic phenotype may be more amenable to established muscle-selection paradigms, such as the COL-CAP concept. Nevertheless, overall clinical treatment outcomes did not differ significantly between patients with and without tremulous/jerky movements, suggesting that the presence of a dynamic component alone may not be sufficient to explain variability in treatment response.

The following limitations of our study should be acknowledged. First, the retrospective nature of the study and the relatively limited number of observations in the subgroup analyses may reduce the robustness of the findings and warrants cautious interpretation. Second, although the semiquantitative assessment of dystonic EMG activity offers practical advantages in a clinical setting, it may provide less precise electrophysiological characterization than fully quantitative methods. Furthermore, because the evaluators were not blinded to the clinical phenotype, potential observer-related bias cannot be excluded, and the precision and reproducibility of this measure may be limited. Third, although the total BoNT dose and the number of injected muscles were not significantly associated with the patient-reported outcome, the potential influence of the muscle-selection strategy on the observed associations cannot be entirely excluded. Fourth, as patients rated the perceived effect of the preceding injection cycle at the end of that cycle, recall bias cannot be excluded. Fifth, we acknowledge that the absence of a standardized post-treatment clinical assessment limited the objective evaluation of treatment response.

## 5 Concluding remarks

The results of the present study support the distinction between tremulous/jerky-predominant and posture-predominant phenotypes of CD. The tremulous/jerky phenotype may be characterized by longer disease duration and less pronounced abnormal posturing, and portable EMG recordings may provide complementary information regarding dystonic muscle activity in this phenotype. In contrast, established muscle-selection paradigms, including the COL-CAP concept, may remain relevant for muscle selection in posture-predominant forms. These findings warrant confirmation in larger, preferably multicentric, prospective studies.

## Data Availability

The raw data supporting the conclusions of this article will be made available by the authors, without undue reservation.
